# Panton-Valentine Leucocidin Proves Direct Neuronal Targeting and Its Early Neuronal and Glial Impacts a Rabbit Retinal Explant Model

**DOI:** 10.3390/toxins10110455

**Published:** 2018-11-04

**Authors:** XuanLi Liu, Michel J Roux, Serge Picaud, Daniel Keller, Arnaud Sauer, Pauline Heitz, Gilles Prévost, David Gaucher

**Affiliations:** 1 Université de Strasbourg, Hôpitaux Universitaires de Strasbourg, Fédération de Médecine Translationnelle de Strasbourg, EA7290 Virulence Bactérienne Précoce, Institut de Bactériologie, F-67000 Strasbourg, France; xuanli.liu2@etu.unistra.fr (X.L.); dkeller@unistra.fr (D.K.); david.gaucher@chru-strasbourg.fr (D.G.); 2 Department of Translational Medicine and Neurogenetics, Institut de Génétique et de Biologie Moléculaire et Cellulaire, CNRS UMR_7104, Inserm U 964, Université de Strasbourg, F-67404 Illkirch, France; mjroux@igbmc.fr; 3 Sorbonne Université, INSERM, CNRS, Institut de la Vision, 17 rue Moreau, 75012 Paris, France; serge.picaud@inserm.fr; 4 Hôpitaux Universitaires de Strasbourg, Service d’Ophtalmologie du Nouvel Hôpital Civil, F-67000 Strasbourg, France; arnaud.sauer@chru-strasbourg.fr (A.S.); pauline.heitz@chru-strasbourg.fr (P.H.)

**Keywords:** Panton-Valentine leukocidin, apoptosis, Müller cell activation, microglial cell activation, retinal explant

## Abstract

Panton-Valentine leukocidin (PVL) retinal intoxication induces glial activation and inflammatory response via the interaction with retinal neurons. In this study, rabbit retinal explant was used as a model to study neuronal and glial consequences of PVL intoxication. Retinal explants were treated with different concentrations of PVL. PVL location and neuronal and glial changes were examined using immunohistochemistry. Some inflammatory factors were quantified using RT-qPCR at 4 and 8 h. These results were compared with those of control explants. PVL co-localized rapidly with retinal ganglion cells and with horizontal cells. PVL induced Müller and microglial cell activation. Retinal structure was altered and some amacrine and microglial cells underwent apoptosis. Glial activation and cell apoptosis increased in a PVL concentration- and time-dependent manner. IL-6 and IL-8 expression increased in PVL-treated explants but less than in control explants, which may indicate that other factors were responsible for glial activation and retinal apoptosis. On retinal explants, PVL co-localized with neuronal cells and induced glial activation together with microglial apoptosis, which confirms previous results observed in in vivo model. Rabbit retinal explant seems to be suitable model to further study the process of PVL leading to glial activation and retinal cells apoptosis.

## 1. Introduction

Bacterial endophthalmitis is an acute ocular infection and often results in poor visual outcomes [1]. The severity of bacterial endophthalmitis is related to virulent infecting strains [2]. *Staphylococcus aureus* is a virulent bacterium frequently found in endophthalmitis cases. The toxins secreted by *S. aureus* are associated with its virulence [3]. The toxins are offensive weapons of *S. aureus*, which attack directly the immune cells and other host cells [4,5]. Analyzing the effects of toxins on the retina could reveal the mechanism by which virulent factors aggravate bacterial endophthalmitis. 

*S. aureus* isolated from human can produce five leukotoxins: two gamma-hemolysins (HlgA/HlgB and HlgC/HlgB), Panton-Valentine leukocidin (PVL), leukocidin ED (LukED), and leukocidin AB (LukAB) [6]. Leukotoxin is composed of two distinct proteins: class S (31–32 kDa) and class F (33–34 kDa) components. The class S component binds to membrane receptors, which allows secondary interaction of the F component. Unaccompanied class S or F proteins do not produce an effect on targeted cells [7]. The PVL gene is present in most community-associated methicillin-resistant *S. aureus*, which is known for its virulence [8]. Horizontal transfer of this gene has been observed, and the percentage of PVL-carrying *S. aureus* has been continuously increasing [9]. PVL-encoding *S. aureus* strains are associated with necrotic infections [10], and, in some rare cases, could cause septic shock after furuncles and severe pneumonia [11]. PVL alone can also cause severe ocular inflammation [12,13,14]. In a PVL-induced endophthalmitis rabbit in vivo model, we previously demonstrated that PVL co-localized with retinal ganglion cells (RGCs) and caused glial cell activation, as well as some microglial apoptosis. Inflammation was also triggered following a PVL infection, as IL-6 and nitrotyrosine increased after intravitreal PVL injection [15].

PVL employs human and rabbit C5a receptors (C5aR) to bind target cells and exert its cytotoxicity [16]. PVL does not recognize murine C5aR, as it exhibits different sequences of amino acids in its second extracellular loop [6]. This preference of animal species is a limiting factor for research on PVL. To resolve this problem, a C5aR humanized mouse was developed. However, the neutrophils of this C5aR humanized mouse have a reduced sensitivity to PVL, because of the different CD45 protein that is a receptor for LukF-PV [17]. This murine model is therefore not widely used. Even if rabbit seems to be a better model than mouse, its utilization in experiment remains limited for ethical reasons. It is necessary to establish an in vitro model to study PVL, which would allow performing more experiments with fewer animal sacrifices.

Primary neuron culture from the dissociated retina is time- and animal-consuming and expensive. It is also difficult to isolate rabbit retinal ganglion cells by the proved method immunopanning due to lack of commercial kits or antibodies [18]. Retinal explants are an alternative to dissociated primary cell culture. It maintains the neurons in situ and in contact with other cells and the extracellular matrix and provides an easily controlled environment. Lacking of retinal and choroidal blood supply, retinal explant can eliminate the possible potential disturbance of myeloid cells in the blood circulation and the effects of blood–ocular barrier breakdown [19].

The purpose of this study was to ascertain that retinal explant can be used as an ex vivo model for studying PVL intoxication and its early consequences on retinal neurons and glia. In this model, as in the previous in vivo model [15], PVL co-localized rapidly with RGCs and induced Müller and microglial cell activation. Moreover, glial activation and cell apoptosis increased in a PVL concentration- and time-dependent manner. Although some discrepancies between the two models have been noticed (e.g., PVL colocalizing with horizontal cells, amacrine cells apoptosis, and lack of IL-6 increase), rabbit retinal explant seems to be a suitable model to further study the process of PVL leading to glial activation and retinal cells apoptosis.

## 2. Results

### 2.1. PVL Co-Localized with RGCs and Horizontal Cells.

**Figure 1:**
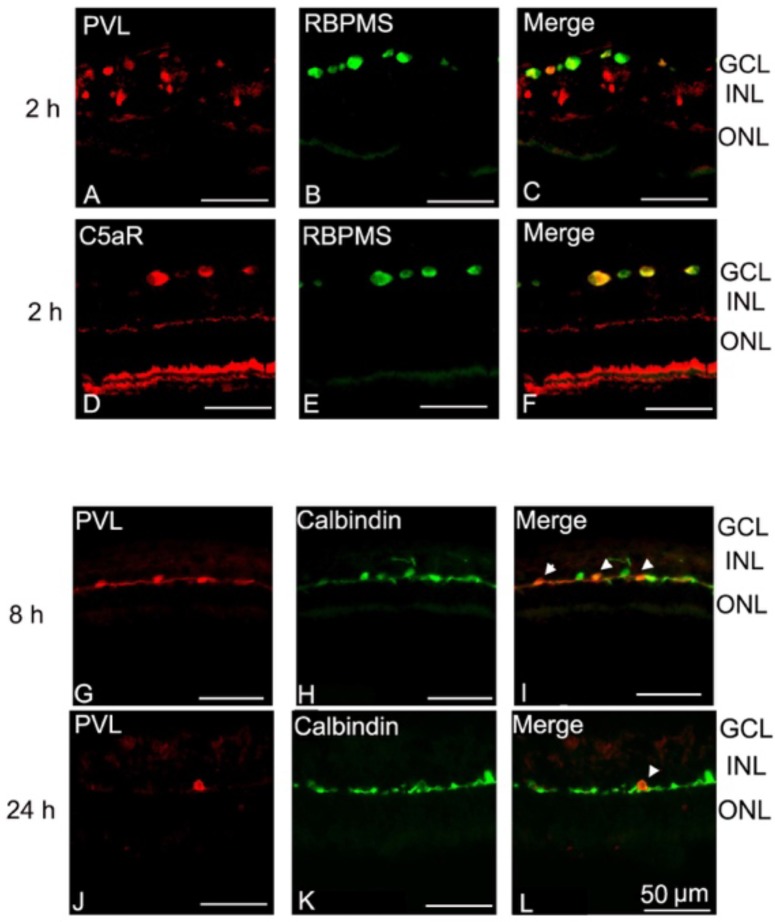
PVL co-localized with RGCs and then with horizontal cells. RGCs co-localized with C5aR immunoactivity. PVL (labeled by anti-LukS-PV, red fluorescence) was located in GCL and INL (**A**) 2 h after PVL treatment. The double-immunohistochemistry showed that PVL co-localized with RGCs labeled with an anti-RBPMS antibody (green fluorescence) (**A**–**C**) in GCL. The C5aR immunoactivity was discovered in GCL and co-localized well with RGCs (**D**–**F**) in another retinal explant 2 h after PVL treatment. After 8 h of PVL treatment in retinal explant, the immunofluorescence of PVL in INL became less (**G**,**J**). PVL co-localized with some anti-Calbindin positive cells (green fluorescence) in the outer limit of INL 8 h (**G**–**I**) and 24 h (**J**–**L**) after PVL treatment. According to the horizontal processes and the location in the outer limit of INL, those PVL-positive cells were horizontal cells. Abbreviations: RBPMS, RNA-binding protein with multiple splicing; GCL, ganglion cell layer; ONL, outer nuclear layer.

**Figure 2:**
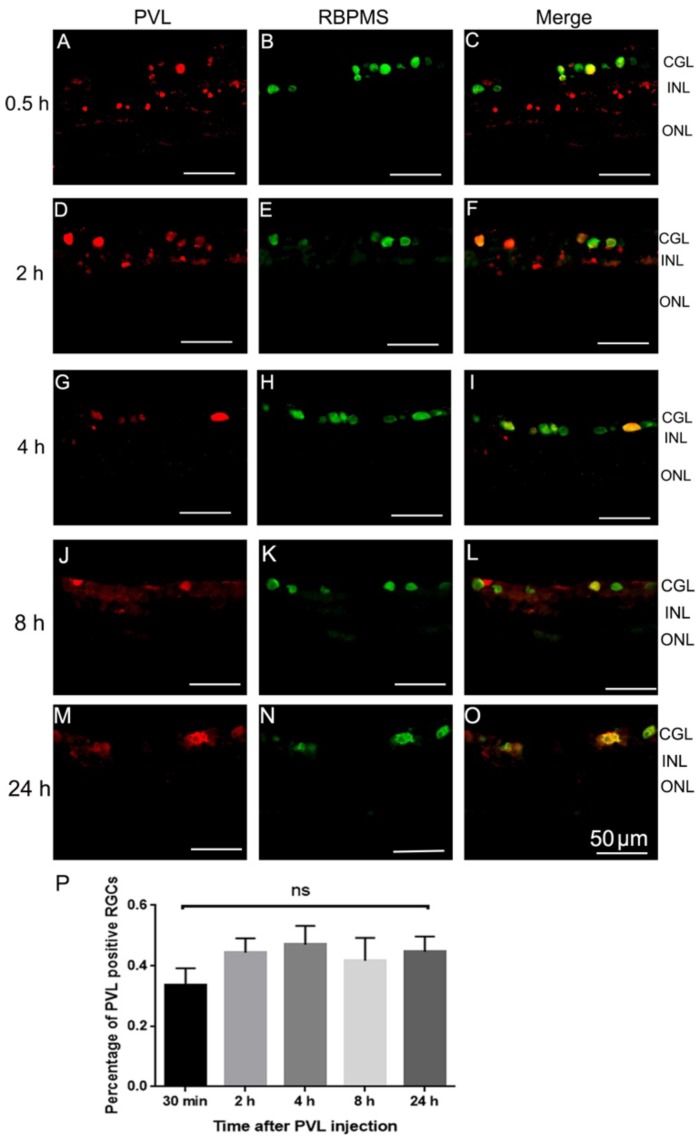
The rate of PVL-positive RGCs did not change from 30 min to 24 h. To better understand the tendency of PVL fixation on RGCs, we measured the rate of PVL-positive RGCs compared to all the RGCs. PVL (red fluorescence) (**A**,**D**,**G**,**J**,**M**) co-localized with some RGCs labeled with an anti-RBPMS antibody (green fluorescence) (**R**,**E**,**H**,**K**,**N**) in the vertical retinal sections. The mean (± SEM) rates of PVL-positive RGCs were 33.7% ± 5.5%, 44.3% ± 4.7%, 47.0% ± 6.2%, 42.0% ± 4.0%, and 45% ± 3.1% for 30 min (**A**–**C**,**P**), 2 h (**D**–**F**,**P**), 4 h (**G**–**I**,**P**), 8 h (**J**–**L**,**P**), and 24 h (**M**–**O**,**P**) after PVL treatment, respectively. No significant difference was observed for the rate of PVL-positive RGCs from 30 min to 24 h (*p* > 0.05, P). Abbreviations: ns, not significant.

After being deposited on the retinal explant, PVL co-localized with RGCs in the retinal section (Figure 1A–C). RGCs also co-localized with C5aR immunoactivity (Figure 1D–F). PVL co-localized with some horizontal cells at 8 and 24 h after PVL treatment (Figure 1G–L). The percentage of PVL-positive RGCs did not significantly change and was around 40% from 30 min to 24 h after PVL treatment (*p* > 0.05, Figure 2A–P). 

PVL immunoreactivity was also observed in some cells in the inner part of the inner nuclear layer before 4 h (INL; Figure 1A–C and Figure 2A–I). The cell type of those cell was not clearly identified. The immunolabeling showed that they were not cholinergic amacrine cells, AII amacrine cells, or calbindin-positive bipolar cells (Figure S1).

### 2.2. Müller and Microglial Cells Were Dramatically Activated Early by PVL in a Concentration- and Time-Dependent Manner

**Figure 3:**
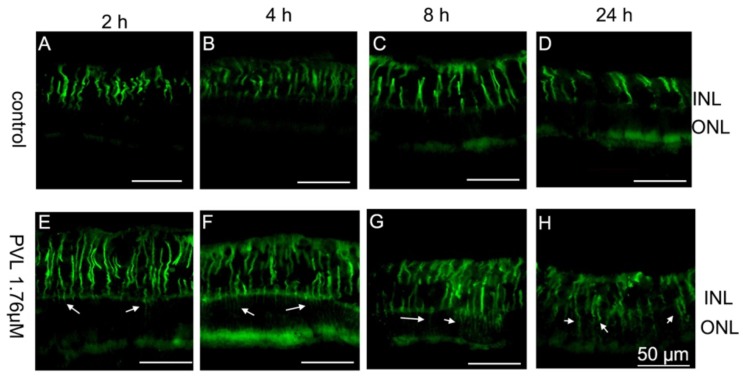
Müller cells were activated in PVL-treated explants. In control explants (**A**–**D**), Müller cell processes were regularly arranged in the inner part of the retina and no process was observed in ONL from 2 to 24 h. In PVL-treated explants, Müller cells showed an abnormal expression of GFAP in ONL, and the outer plexiform layer (OPL) was abnormally noticed by increase of GFAP staining (arrow) (**E**–**G**) from 2 to 8 h after PVL treatment, demonstrating the abnormal glial reactivity. At 24 h, the anti-GFAP labeling was visible in the whole retina, from the inner to the outer part, and GFAP staining of OPL was disrupted (arrow) (**H**).

**Figure 4:**
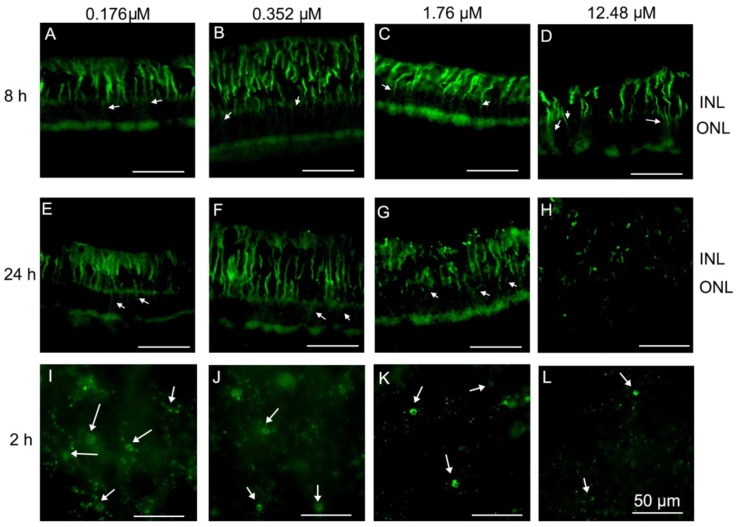
In PVL-treated explants, Müller and microglial cells were activated in a concentration- and time-dependent manner: (**A**–**H**) vertical retinal sections; and (**I**–**L**) whole retinal mounts. Müller cells (labeled with anti-GFAP) expressed abnormally GFAP in the ONL and OPL was visible by abnormal expression of GFAP 8 h after PVL treatment, which increased from 0.176 to 1.76 µM PVL (arrow) (**A**–**C**). Müller cells showed a dissociated arrangement when treated with 12.48 µM PVL for 8 h (arrow) (**D**). Müller cells showed an abnormal extension in the ONL and OPL was visible by abnormal expression of GFAP 24 h after treatment with 0.176 and 0.352 µM PVL (arrow) (**E**,**F**). Müller cells appeared damaged, and the retinal structure was dissociated when treated with 1.76 (arrow) (**G**) and the processes of Müller cells were destroyed into pieces after treatment of 12.48 µM (arrow) (**H**) PVL for 24 h. Two hours after culture, microglial cells (labeled with FITC-tagged GSAI-B4) began to dissociate and lose their processes under 0.176 µM PVL treatment (arrow) (**I**). Their processes were dissolved under 0.176 µM and 0.352 µM PVL treatment (arrow) (**I**,**J**) and disappeared with 1.76 µM PVL treatment (arrow) (**K**), and their soma became smaller when treated with 12.48 µM PVL (arrow) (**L**). Abbreviations: FITC, Fluorescein isothiocyanate; GSAI-B4, Griffonia Simplicifolia I Isolectin B4.

Müller cells extended their processes through the whole retina to provide architectural support to retinal neurons. The hallmark of retinal Müller cell activation is the rapid upregulation of glial fibrillary acidic protein (GFAP) following acute retinal injury [20]. In control explants, Müller cells were regularly arranged on the inner side of the retina from 2 to 24 h after culture (Figure 3A–D). Müller cells showed an abnormal extension in the outer nuclear layer in PVL-treated explants, which increased overtime after PVL treatment (Figure 3E–H) and from low to high PVL concentrations (Figure 4A–H). Dissociation of Müller cell organization was observed in retinal explants treated with low concentration of PVL (Figure 3H and Figure 4G), while the retinal architecture was highly damaged when treated with higher concentration of PVL (Figure S2). This retinal disorganization may be related to the dysfunction of Müller cells.

**Figure 5:**
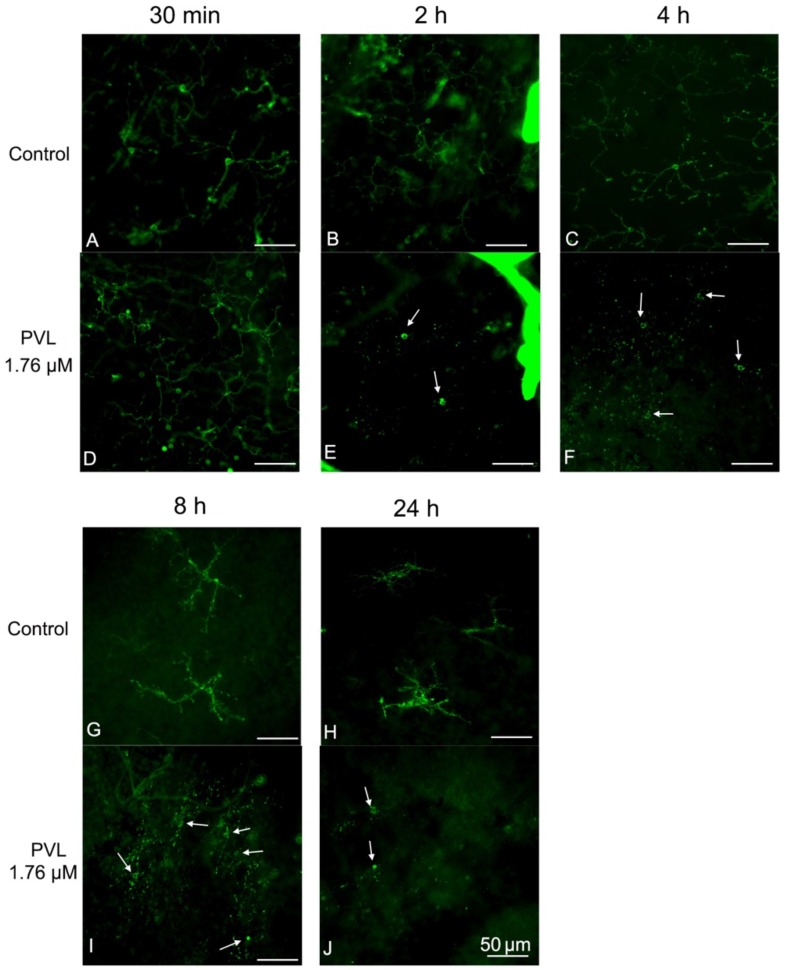
Microglial cells were activated 2 h after PVL treatment: (**A**–**J**) images of the whole retinal mount. Microglial cells were labeled with FITC-tagged GSAI-B4. In control explants, microglial cells were normal with ramified dendrites before 4 h (except for a very few cells which could be considered as ameboid) (**A**–**C**) and retracted their processes 8 and 24 h after culture (arrow) (**G**,**H**). In contrast, microglial cells were normal with ramified dendrites at 30 min (**D**) in PVL-treated explants (1.76 µM), but were drastically dissociated with their dendrites and transformed into amoeboid forms from 2 h to 24 h under 1.76 µM PVL treatment (arrows) (**E**,**F**,**I**,**J**).

Microglial cells could demonstrate a broad range of morphological changes at different activated stages, including an enlargement of the soma, retraction and shortening of processes, and transformation into an amoeboid form, a round cell soma without processes [21]. In control explants, all the microglial cells began to retract their processes 8 h after culture, enlarged their soma and retracted their processes 24 h after culture (very few cells seemed to be amoeboid before 8 h) (Figure 5G,H). In PVL-treated explants, the processes of the microglial cells were all dissociated from the soma and microglia cells were transformed into the amoeboid form, losing all their processes as early as 2 h after PVL treatment (Figure 5E,F,I,J). Microglial cells were dramatically activated regardless of concentration of PVL (Figure 4I–L).

### 2.3. Some Microglial and Amacrine Cells Underwent Apoptosis in PVL-Treated Explants.

**Figure 6:**
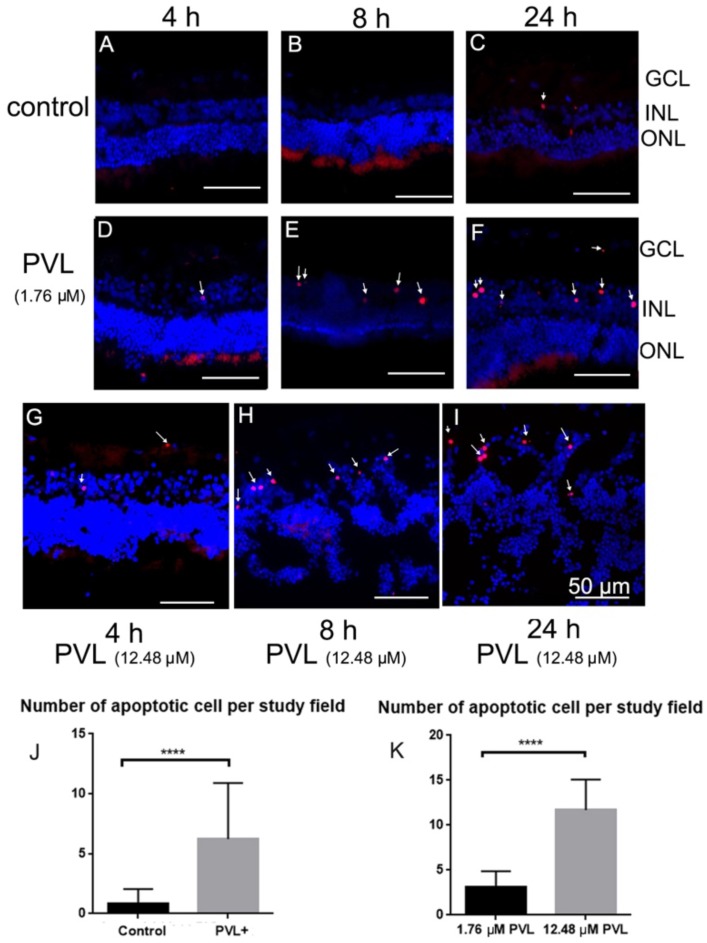
In PVL-treated explants, TUNEL-positive cells were found and increased in a concentration- and time-dependent manner. In control explants, no TUNEL-positive cells were detected 4 (**A**) or 8 h (**B**) after culture. However, a few TUNEL-positive cells were detected 24 h after culture (arrow) (**C**). In PVL-treated explants (1.76 and 12.48 µM), TUNEL-positive cells were discovered mostly in INL, and the number of TUNEL-positive cells was increased from 4 to 24 h after treatment (arrow) (**D**–**I**). In 12.48 µM PVL-treated explants, retinal cell layers labeled with Hoechst dye were dissociated and became not distinguishable, and the retinal structure was damaged 8 (**H**) and 24 h (**I**) after treatment. The number of TUNEL-positive cells in PVL-treated explants was increased compared with control explants (**** *p* < 0.0001) (**J**). It was also significantly increased in 12.48 µM PVL-treated explants (**H**,**I**) compared with 1.76 µM PVL-treated explants (**E**,**F**,**K**) (**** *p* < 0.0001).

In control explants, a few terminal deoxynucleotidyl transferase dUTP nick end labeling (TUNEL)-positive cells were only found 24 h after culture (Figure 6A–C and Table 1). In PVL-treated explants, several TUNEL-positive cells were observed at 4 h after PVL treatment (Figure 6D,G) and their number increased at 8 and 24 h after PVL treatment (Figure 6E–G,I). In total, PVL-treated explants exhibited significantly more TUNEL-positive cells than control explants (**** p < 0.0001, Figure 6J). The number of TUNEL-positive cells increased with the augmentation of PVL concentration and the time following PVL treatment. For instance, 24 h after high concentration of PVL treatment, the number of apoptotic cells was increased nearly fourfold compared with explants treated with low concentration of PVL (Table 1). 

**Figure 7:**
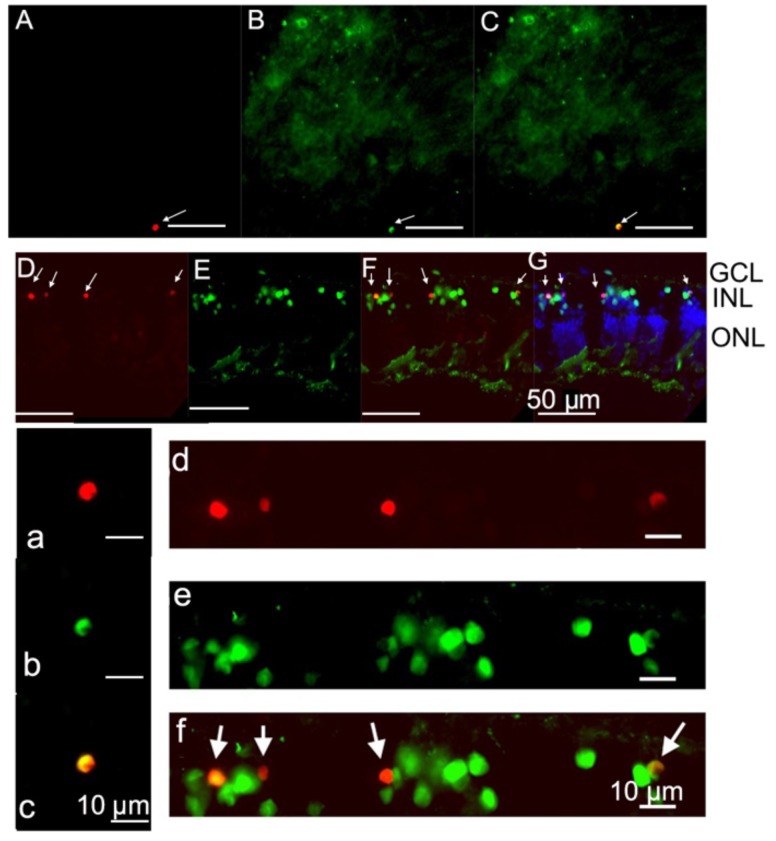
Some microglial and amacrine cells were identified as TUNEL-positive cells: (**A**–**C**) images of the whole retinal mount. The double-labeling between TUNEL and FITC-tagged GSAI-B4 showed that some microglial cells were TUNEL-positive cells (**A**–**C**) (enlarged images (**a**–**c**)). In vertical retinal sections, the majority of TUNEL-positive cells were discovered in the INL (arrow) (**D**). Anti-Pax 6 antibody labels all the amacrine cells in retina. The double-labeling between TUNEL and anti-Pax 6 showed that the TUNEL-positive cells in INL were amacrine cells in vertical retinal sections (**D**–**G**) (enlarged images (**d**–**f**)).

Double-labeling using TUNEL and antibodies labeling retinal cells was employed to identify the type of retinal cells which underwent apoptosis. It was shown that some microglial cells (Figure 7A–C,a–c) and some amacrine cells within the INL were TUNEL-positive (Figure 7D–G,d–f). RGCs and calbindin-positive bipolar or horizontal cells were not TUNEL-positive (Figure S3). 

### 2.4. In PVL-Treated Explants, the Expression of Some Inflammatory Factors Was Not as Increased as in Control Explants

**Figure 8:**
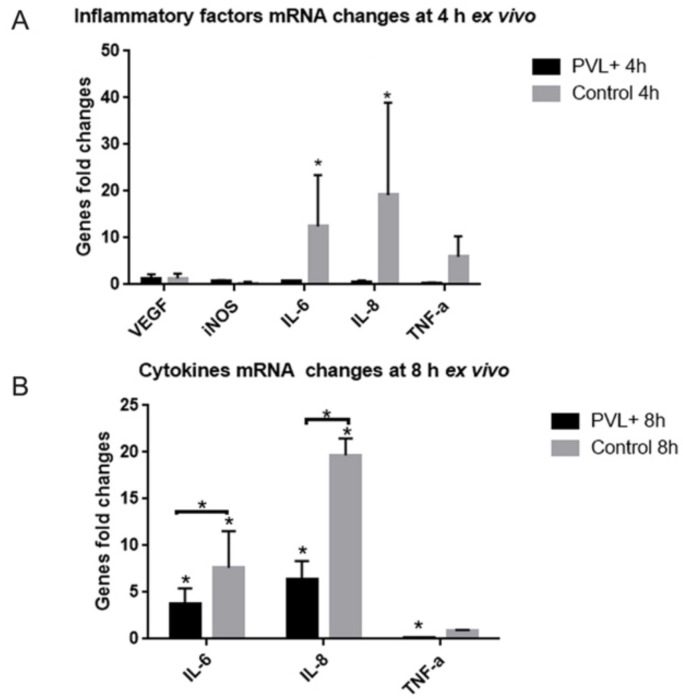
The expression of some inflammatory factors was lower in PVL-treated explants than in controls. RT-qPCR was used to analyze expression of some inflammatory factors (VEGF, iNOS, IL-6, IL-8 and TNF-α) in explants 4 and 8 h after culture. Four hours after culture, control explants had significantly increased the expression of IL-6 (**A**) (12.42 ± 6.40-fold change, * *p* < 0.05) and IL-8 (19.13 ± 11.43-fold change, * *p* < 0.05), whereas PVL-tested explants did not significantly increase the expression of IL-6 and IL-8. At 8 h after culture, control and PVL-tested explants had significantly increased IL-6 and IL-8 mRNA expressions, whereas control explants expressed more than PVL-treated explants: IL-6, 7.63 ± 2.25 vs. 3.70 ± 0.98 (**B**) (* *p* < 0.05); IL-8, 19.66 ± 1.05 vs. 6.36 ± 1.16 (* *p* < 0.05). The expression of TNF-α was significantly decreased in PVL-tested explants (0.15 ± 0.02-fold change, * *p* < 0.05), but not changed in control explants (0.89 ± 0.04-fold change, *p* > 0.05). The expression of VEGF and iNOS were not changed in all the explants.

RT-qPCR was used to analyze expression of some inflammatory factors (vascular endothelial growth factor (VEGF), inducible nitric oxide synthase (iNOS), IL-6, IL-8 and TNF-α) in explants 4 and 8 h after culture. Four hours after culture, control explants increased significantly the expression of IL-6 and IL-8. Conversely, IL-6 and IL-8 expression did not significantly increase in PVL-treated explants (Figure 8A). 

Eight hours after culture, PVL-treated explants had significantly increased IL-6 and IL-8 mRNA expression, but less than in the control explants (*p < 0.05, Figure 8B). In PVL-treated explants, TNF-α expression significantly decreased, which was not the case for control explants. 

## 3. Discussion 

In retinal explants, PVL co-localized rapidly with RGCs and then with horizontal cells. PVL induced the activation of Müller and microglial cells together with amacrine and microglial cell apoptosis in a concentration- and time-dependent manner.

PVL is a virulent leukotoxin of *S. aureus* that targets human or rabbit neutrophils, macrophages and monocytes. It was also demonstrated that PVL has tropism towards neurons [22]. We recently showed that intravitreal injection of PVL induces severe retinal inflammation after co-localizing with RGCs, which demonstrates that PVL could induce inflammation in retinal tissue after targeting neurons [15]. To study the interaction between neurons and glial cells, we decided to create an ex vivo model, using retinal explant. Retinal explant presents some advantages compared to the in vivo model. Retinal explant is reproducible and easy to obtain. It is devoid of blood supply and, without the presence of leukocytes, we can eliminate the interference of leukocytes in analyzing PVL effects on retinal neurons. From an ethic point view, it allows performing more experiments with less rabbit sacrifice.

In the present ex vivo study, PVL rapidly co-localized with RGCs and later with horizontal cells. Some cells in the INL were PVL-positive, but we did not clearly identify the cell type of those cells. Those cells were not bipolar, horizontal, starburst, or AII amacrine cells. Interestingly, some amacrine cells in INL underwent apoptosis a few hours after PVL treatment. Those apoptotic amacrine cells were neither starburst nor AII amacrine cells (Figure S3). It is possible that those PVL-positive cells in INL are a subpopulation of amacrine cells, probably the same subgroup of amacrine cells that undergo apoptosis.

As in in vivo model, RGCs were the major cells targeted by PVL. However, more cellular subtypes colocalized with PVL in retinal explant (Table 2). This might be due to lower resistance of diffusion in explants. The inner and outer plexiform layers are the sites of highest resistance to molecular diffusion in retina [23].The retinal explant lacks a blood supply and the retinal vasculature rapidly shrinks. This might decrease the capacity of diffusion resistance from the inner plexiform layer. Consequently, PVL may diffuse more easily through the retina and reach the INL (i.e., horizontal cells).

PVL caused apoptosis of a subpopulation of amacrine cells in PVL-treated explants. PVL could induce an increase of intracellular calcium and glutamate release from neuronal cells [22]. The amacrine cells express ionotropic glutamate receptors. Excessive activation of ionotropic glutamate receptors could lead to amacrine cell death [24]. The PVL-positive cells were RGCs and some neuronal cells in the INL, which might release excessive glutamate and induce apoptosis in amacrine cells.

We showed that Müller cells were activated in a PVL concentration- and time-dependent manner. This Müller cell reaction was also associated with retinal structural damage. Significant destruction of the retinal structure occurred 8 h after PVL treatment and might be due to disfunction of Müller cells. Müller cells are the primary cells responsible for K^+^ and fluid influx regulation in the retina [25]. In retinal inflammation, Müller cells are activated and transformed into gliosis, which results in a significant decrease in potassium and water channel protein expression [26]. Müller cells swell and the retinal fluid absorption function decreases, leading to retinal edema and degeneration [27].

Microglial cell activation is a typical early phenomenon in response to retinal injuries and inflammation before retinal cell death [28]. Activated microglial cells demonstrate various phenotypes with a different degree of stimuli and could undergo apoptosis by overactivation [29]. Microglial cells are very sensitive to changes of environment. They could be activated by disturbance of the balance of excitatory and inhibitory stimuli from the microenvironment around [30,31,32]. In control explants, microglial cells were slightly activated at 8 and 24 h after culture. Microglial cells were dramatically activated even 2 h after PVL treatment; some of them were apoptotic, which might be because of the overaction incited by PVL.

In PVL-treated explants, the increase of IL-6 and IL-8 expression was late and mild compared to controls. Even if these inflammatory factors were not increased, Müller and microglial cells were dramatically activated. This could demonstrate that glial activation is not related to the increase of IL-6, which was recently observed in vivo model following PVL intoxication [15]. IL-6 increase could be a consequence of glial activation. However, in PVL-treated explants, it is possible that Müller and microglial cells are too altered (Figure 4) to express inflammatory factors. IL-6 and IL-8 are probably not the early and key factors for PVL-inducing glial activation and inflammation in retina. Further studies should try to identify the causative factors especially released by retinal neurons such as neurotransmitters or some small inflammatory molecules. 

In conclusion, the retinal ganglion cells are the principal targeted cells by PVL, in both in vivo and ex vivo models of PVL retinal intoxication. While retinal explant showed limited correspondence with the in vivo model concerning the inflammatory response, glial activation and microglial apoptosis were similar in both models. Retinal explant can be a useful tool to further study the mechanisms leading to glial activation and retinal cell apoptosis. This model may facilitate the exploration of neuronal response to PVL using intracellular calcium imaging [33] and the tests of new therapies against PVL retinal intoxication. 

## 4. Materials and Methods

### 4.1. PVL Purification

PVL (LukS-PV/LukF-PV) was purified as described in a previous study [34] by affinity chromatography on glutathione-Sepharose 4B followed by cation-exchange fast-performance liquid chromatography after removal of glutathione S-transferase tag with Precision Protease (GE Healthcare, Villacoublay, France). Preparation homogeneity was assessed by radial gel immunoprecipitation and SDS-polyacrylamide gel electrophoresis before storage at −80 °C.

### 4.2. Retinal Explant Preparation and Organotypic Culture

The animal experiments were approved by the Ministère de l’Education nationale, de l’Enseignement supérieur et de la Recherche, France (Apafis N°4986, date of approval: 2 February 2017). The surgical procedure was performed in accordance with the guidelines in the laboratory of the Association for Research in Vision and Ophthalmology.

Retinal explant requires swift extraction of the retina and retinal flat mounting on a hydrophilic membrane with minimum disturbance of the tissue. Briefly, pigmented rabbits (Bleu de Champagne) aged 6 months and weighing 2.5–3 kg were anesthetized by a lumbar intramuscular injection of ketamine, 20 mg/kg (Virbac, Carros, France) and xylazine, 3 mg/kg (Bayer Healthcare, Puteaux, France), followed by a lethal intravenous injection of 2 mL Pentobarbital Dolethal^®^ (Vetoquinol, Lure, France) through a 22-Gauge catheter inserted in the marginal auricular vein. The eyes were immediately enucleated after euthanasia and immersed in ice-cold CO_2_-independent medium (Gibco, Life technologies, Carlsbad, CA, USA). Eyes were transported to aseptic condition. Each eyeball was immersed in disinfection medium (Pursept A, xpress, Norderstedt, Germany) and washed with cold i-CO_2_ medium. Under a stereomicroscope, the eye globes were dissected and the posterior segment was cut into four 7 mm × 7 mm pieces avoiding to cut out visible blood vessels and myelinilized retinal parts. The choroid was teared away from the sclera and the optic nerve was cut with fine scissor. The neuroretina was gently detached from the pigment epithelium by tearing the choroid off. 

The retina was dropped with photoreceptor layer facing down on membrane which was inserted into Transwell^®^ culture dishes (Corning Inc, Corning, NY, USA). After the retinal flat-mounting, 2 mL culture medium, neurobasal-A (Gibco, Life technologies, Carlsbad, CA, USA) supplemented with 1% antibiotic–antimycotic mixture, was added into the culture well. The culture medium level was maintained in contact with the support membrane beneath the explant. The retinal explants were incubated at 37 °C with 5% CO_2_ in a humidified atmosphere.

### 4.3. PVL-Treated Explants and Control Explants

Series of PVL concentrations (0.176, 0.352, 1.76 and 12.48 µM) were prepared and applied to 3 or 6 (for 1.76 µM PVL) different explants at each time point (30 min, 2 h, 4 h, 8 h and 24 h). Our previous study used PVL (3 µg/50 mL), equal to 1.76 µM. Thus, 1.76 µM PVL was used as reference concentration. A 10 μL droplet of PVL diluted in culture medium was deposited on the surface of nerve fiber layer in each tested explant after retina flat-mounting. An equivalent volume of culture medium was deposited onto the surface of control explants. Retina explants were collected and immediately fixed by 4% (*w/v*) paraformaldehyde. Three explants treated by 1.76 µM PVL were immediately frozen at −80 °C for RT-qPCR at each time point (4 and 8 h).

### 4.4. Tissue Processing

The explants were fixed for 1 h in 4% (*w*/*v*) paraformaldehyde and then embedded successively in 10% (*w*/*v*) and 20% (*w*/*v*) sucrose and stored in 30% (*w*/*v*) sucrose overnight at 4 °C. The fixed retinal explants were divided and immersed in optimal cutting temperature compound (Sakura Finetek, Torrance, CA, USA) for cryosections, or stored in plastic tube (0.5 mL) directly at −80 °C for retinal whole-mounts. Retinal cryosections of 8 μM were cut and mounted on a Super Frost^TM^ Plus microscope slides (Thermo Fisher Scientific, Rockford, IL, USA) and stored at −20 °C. 

### 4.5. Fluorescent Immunostaining 

Retinal sections were permeabilized in 0.05% (*v*/*v*) TritonX-100 for 1 h and then were blocked with 10% (*v*/*v*) donkey serum (Sigma-Aldrich, St. Louis, MO, USA) for 1 h. Retinal sections were incubated with primary antibodies (Table 3) at 4 °C overnight in a humidity chamber. After washing with PBS × 1 for 3 times, retinal sections were then incubated for 1 h at room temperature with fluorescent secondary antibodies (Table 3). Some sections were continually incubated in TUNEL mixed solution for another 1 h. After washing with PBS × 1 for 3 times, sections were counter-stained with Hoechst 33258 and mounted in 10% (*v*/*v*) Mowiol^®^ solution (Polysciences, Eppelheim, Germany). 

Retina whole mounts were permeabilized 0.2% (*v*/*v*) TritonX-100 for 30 min. The retina whole mounts were incubated in conical small wells and transferred with 3 mL pipette. The staining steps were the same as those of immunoistochemistry section. Finally, the retinal whole mounts were mounted on microscope slides and covered with thinner and smaller cover slides in 10% (*v*/*v*) Mowiol^®^ solution. Images of fluorescent sections and whole mounts were obtained using an epifluorescence Olympus BX60 microscope connected to a Hamamatsu C11440 digital camera.

### 4.6. Cell Counting

Different microscope fields (266 µm × 266 µm) of retinal immunofluorescent images were captured by the camera. PVL-positive retinal cells were evaluated by double immunohistochemistry: PVL-positive cells were double-labeled by PVL and retinal cell-specific markers. The percentages of PVL-positive RGCs at each time point (30 min, 2 h, 4 h, 8 h and 24 h with 1.76 µM PVL) were established by mean mounts of 3 explants (each explant has five study fields randomly captured by camera). For TUNEL positive cells count, five different microscope fields were analyzed for each explant, three different explants for each time point and PVL concentration. The mounts of TUNEL positive cells in each time points and concentration were established by mean mounts of all study fields. 

### 4.7. RNA Extraction 

TRIzol reagent (Sigma, Saint-Louis, MO, USA) was added into tubes contained frozen retina. The retinas were passed through 23-Gauge needle then 26-Gauge needle several times to be homogenized. Total RNA was isolated using TRIzol reagent according to the manufacturer’s instructions. The total RNA concentration was quantified with spectrophotometry (NanoDrop; Thermo Scientific, Waltham, MA, USA). 

### 4.8. Real-Time RT-qPCR

Ten-microgram RNA aliquots were treated with DNA-free kit DNase treatment (Ambion, Life technologies, Carlsbad, CA, USA) at 37 °C for 30 min according to manufacturer’s instructions. 5 µL of RNA solution after DNase treatment was immediately reverse transcribed (RT) using Superscript First-Strand Synthesis for RT-PCR (Invitrogen, Life technologies, Carlsbad, CA, USA). Diethyl decarbonate (DEPC) (Sigma) treated H_2_O was added to RT mixture (0.5 µL random hexamers (200 ng/mL) and 5 µL total RNA, 1 µL NTP) to achieve a 12 µL volume, and then incubated at 65 °C for 5 min and placed in glass for 2 min. Then, 0.5 µL of 0.1 M DDT, 0.5 µL of transcriptase, 4 µL of First Strand buffer, and 3 µL of sterile H_2_O were added to the mixture. Then, the total mixture was put into a ThermoCycler programmed at 42 °C for 50 min and at 70 °C for 15 min. The cDNA was diluted 3 times with DEPC treated H_2_O. Then, 5 µL of diluted cDNA, 10 µL SYBR Green mix (LightCycler 480 SYBR Green I Master, Roche, Basel, Switzerland), 2 µL of forward and reverse primers (100 µm), and 3 µL H_2_O were mixed and put into 96 wells plate. The plate was placed into Real-Time PCR System (Light Cycler 480, Roche, Basel, Switzerland). PCR was programmed as initial denaturation step at 95 °C for 10 min, 45 cycles of amplification (denaturation at 95 °C for 15 s, annealing at 60 °C for 20 s, extension at 72 °C for 15 s), and melting curve analysis (60–95 °C, increment at 0.3 °C). The specificity of PCR products was verified according to one melting curve peak and one band in agarose gel electrophoresis. Products of RTs without reverse transcriptase were used as controls to ascertain no significant DNA contamination. 

The primers were designed to have Tm around 60 °C by using Primer3 input (Version 4.1.0, http://primer3.ut.ee/). The sequences of primers are shown in Table 4. β-actin was used as reference gene and target genes were normalized using this reference gene. The method ∆Ct was used to calculate relative quantification between control explants and PVL-treated explants. The fold changes were calculated using 2^−∆∆Ct^. The tests were triplicate. The significant changes of every target gene were statistically analyzed using ∆Ct paired t-tests.

### 4.9. Statistical Analysis 

Statistical analysis was performed with GraphPad InStat version 3.10 (GraphPad Software, CA, USA, 2013). Statistical significance was calculated with paired t-tests, unpaired t-tests or ANOVA test. Statistical significance was assumed at *p* < 0.05. 

## Figures and Tables

**Table 1:** The mean number of apoptotic cells per study field.

PVL Concentration	4 h	8 h	24 h
**0 (Control)**	0	0	0.81
**0.176 μM**	0	0.22	2.50
**0.352 μM**	0	1.36	3.67
**1.76 μM**	2.25	2.00	5.00
**12.48 μM**	2.00	6.00	11.67

Table 1 the mean number of apoptotic cells per study field of different explants 4, 8 and 24 h after treatment of different PVL concentrations. The mean number of apoptotic cells was calculated from three different explants and five study fields of each explant.

**Table 2:** Similar and different results obtained in the in vivo model and the ex vivo explant model of PVL retinal intoxication.

Results	Retina in Vivo	Retinal Explant Ex Vivo
**PVL Targeted Cells**	RGCs, Displaced amacrine cells	RGCs, Some cells in the interne nuclear layer, Horizontal cells
**Cell Apoptosis**	Microglial cells	Microglial cells, A subpopulation of amacrine cells
**Effects of Glial Cells**	Activation of Müller cells; Activation and apoptosis of microglial cells	Activation of Müller cells; Activation and apoptosis of microglial cells
**Inflammatory Factors**	IL-6 increased, IL-8 not increased	IL-6 increased, but not as much as in the control group; IL-8 increased, but not as much as in the control group

**Table 3:** List of Specific markers used in the current study. Primary antibodies or lectin.

Target	Antiserum	Source	Concentration
**PVL**	Rabbit anti-LukS-PV polyclonal	EA-7290, Strasbourg, France	2 µg/mL
**C5aR**	Rabbit anti-C5aR polyclonal	Abcam, Cambridge, UK	2 µg/mL
**Ganglion Cells**	Guinea pig anti-RBPMS polyclonal	UCLA Neurobiology, Los Angeles, CA, USA	2 µg/mL
**Starburst Amacrine Cells**	Goat anti-ChAT polyclonal	Chemicon Merck-Millipore, Temecula, CA, USA	20 µg/mL
**Müller Cells**	Mouse anti-GFAP polyclonal	Bio-Rad AbD Serotec, Oxfordshire, UK	2 µg/mL
**Microglial Cells**	FITC-tagged GSAI-B4	Sigma Aldrich, Saint Louis, MO, USA	2 µg/mL
**Amacrine Cells**	Rabbit anti-Pax 6 polyclonal	Abcam	2 µg/mL
**AII Amacrine Cells**	Mouse anti-calretinin monoclonal	Santa Cruz Biotechnology, Heidelberg, Germany	2 µg/mL
**Horizontal Cells**	Mouse anti-calbindin monoclonal	Santa Cruz Biotechnology	2 µg/mL
**Secondary Antibodies**			
**Anti-Rabbit**	Goat and donkey polyclonal Alexa 555nm-conjugated	Life Technologies, Carlsbad, CA, USA	2 µg/mL
**Anti-Goat**	Donkey polyclonal Alexa 488-conjugated	Molecular Probes, Eugene, OR, USA	2 µg/mL
**Anti-Mouse**	Donkey polyclonal Alexa 488-conjugated	Abcam	2 µg/mL
**Anti-Guinea Pig**	Goat polyclonal Alexa 488-conjugated	Abcam	2 µg/mL
**TUNEL**	DNA strand breaks	Roche Life Science, Indianapolis, IN, USA	-
**Nuclei**	Hoechst 33258	Molecular Probes^TM^, Eugene, OR, USA	0.1 µg/mL

**Table 4:** The sequences of primers used this study.

Gene names	Forward Primer	Afterward Primer
**β-actin**	5’-gcgggacatcaaggagaag-3’	5’-aggaaggagggctggaaga-3’
**IL-6**	5’-tcaggccaagttcaggagtg-3’	5’-atgaagtggatcgtggtcgt-3’
**IL-8**	5’-tggctgtggctctcttgg-3’	5’-atttgggatggaaaggtgtg-3’
**TNF-α**	5’-cgtagtagcaaacccgcaag-3’	5’-tgagtgaggagcacgtagga-3’
**VEGF**	5’-cgagaccttggtggacatctt-3’	5’-tgcattcacatttgttgtgct-3’
**iNOS**	5’-ccaagccctcacctacttcc-3’	5’-aactcctccagcacctcca-3’

## Supplementary Materials

The following are available online at http://www.mdpi.com/2072-6651/10/11/455/s1, Figure S1: PVL did not colocalize with cholinergic amacrine cells, AII amacrine cells; or calretinin-positive cells (bipolar and horizontal cells) within 4 h of PVL treatment. Figure S2: Müller cells were activated and retinal structure was destroyed in PVL-treated explants. Figure S3: TUNEL positive cells did not colocalized with RGCs, cholinergic amacrine cells, AII amacrine cells, or calretinin-positive cells (bipolar and horizontal cells) in PVL-treated explants.

## References

[1] Callegan M.C., Engelbert M., Jett B.D., Gilmore M.S. (2002). Bacterial endophthalmitis: Epidemiology, therapeutics, and bacterium-host interactions. Clin. Microbiol. Rev..

[2] Callegan M.C., Gilmore M.S., Gregory M., Ramadan R.T., Wiskur B.J., Moyer A.L., Hunt J.J., Novosad B.D. (2007). Bacterial endophthalmitis: Therapeutic challenges and host-pathogen interactions. Prog. Retin. Eye Res..

[3] Vincenot F., Saleh M., Prévost G. (2008). Les facteurs de virulence de *Staphylococcus aureus*. Revue Francophone des Lab..

[4] Spaan A.N., van Strijp J.A.G., Torres V.J. (2017). Leukocidins: staphylococcal bi-component pore-forming toxins find their receptors. Nat. Rev. Microbiol..

[5] Rooijakkers S.H., Ruyken M., van Roon J., van Kessel K.P., van Strijp J.A., van Wamel W.J. (2006). Early expression of SCIN and CHIPS drives instant immune evasion by Staphylococcus aureus. Cell. Microbiol..

[6] Spaan A.N., Schiepers A., de Haas C.J., van Hooijdonk D.D., Badiou C., Contamin H., Vandenesch F., Lina G., Gerard N.P., Gerard C. (2015). Differential Interaction of the Staphylococcal Toxins Panton-Valentine Leukocidin and gamma-Hemolysin CB with Human C5a Receptors. J. Immunol..

[7] Rd A.F., Torres V.J. (2014). The bicomponent pore-forming leucocidins of Staphylococcus aureus. Microbiol. Mol. Biol. Rev. MMBR.

[8] Vandenesch F., Naimi T., Enright M.C., Lina G., Nimmo G.R., Heffernan H., Liassine N., Bes M., Greenland T., Reverdy M.E. (2003). Community-acquired methicillin-resistant *Staphylococcus aureus* carrying Panton-Valentine leukocidin genes: Worldwide emergence. Emerg. Infect. Dis..

[9] Diep B.A., Gill S.R., Chang R.F., Phan T.H., Chen J.H., Davidson M.G., Lin F., Lin J., Carleton H.A., Mongodin E.F. (2006). Complete genome sequence of USA300, an epidemic clone of community-acquired meticillin-resistant *Staphylococcus aureus*. Lancet.

[10] Lina G., Piemont Y., Godail-Gamot F., Bes M., Peter M.O., Gauduchon V., Vandenesch F., Etienne J. (1999). Involvement of Panton-Valentine leukocidin-producing *Staphylococcus aureus* in primary skin infections and pneumonia. Clin. Infect. Dis. Off. Publ. Infect. Dis. Soc. Am..

[11] Gillet Y., Issartel B., Vanhems P., Fournet J.C., Lina G., Bes M., Vandenesch F., Piemont Y., Brousse N., Floret D. (2002). Association between *Staphylococcus aureus* strains carrying gene for Panton-Valentine leukocidin and highly lethal necrotising pneumonia in young immunocompetent patients. Lancet.

[12] Laventie B.J., Potrich C., Atmanene C., Saleh M., Joubert O., Viero G., Bachmeyer C., Antonini V., Mancini I., Cianferani-Sanglier S. (2013). p-Sulfonato-calix[n]arenes inhibit staphylococcal bicomponent leukotoxins by supramolecular interactions. Biochem. J..

[13] Laventie B.J., Rademaker H.J., Saleh M., de Boer E., Janssens R., Bourcier T., Subilia A., Marcellin L., van Haperen R., Lebbink J.H. (2011). Heavy chain-only antibodies and tetravalent bispecific antibody neutralizing *Staphylococcus aureus* leukotoxins. Proc. Natl. Acad. Sci. USA.

[14] Siqueira J.A., Speeg-Schatz C., Freitas F.I., Sahel J., Monteil H., Prevost G. (1997). Channel-forming leucotoxins from *Staphylococcus aureus* cause severe inflammatory reactions in a rabbit eye model. J. Med. Microbiol..

[15] Liu X., Heitz P., Roux M., Keller D., Bourcier T., Sauer A., Prevost G., Gaucher D. (2018). Panton-Valentine Leukocidin Colocalizes with Retinal Ganglion and Amacrine Cells and Activates Glial Reactions and Microglial Apoptosis. Sci. Rep..

[16] Spaan A.N., Henry T., Van Rooijen W.J., Perret M., Badiou C., Aerts P.C., Kemmink J., De Haas C.J., Van Kessel K.P., Vandenesch F. (2013). The Staphylococcal Toxin Panton-Valentine Leukocidin Targets Human C5a Receptors. Cell Host Microbe.

[17] Tromp A.T., Van Gent M., Abrial P., Martin A., Jansen J.P., De Haas C.J.C., Van Kessel K.P.M., Bardoel B.W., Kruse E., Bourdonnay E. (2018). Human CD45 is an F-component-specific receptor for the staphylococcal toxin Panton-Valentine leukocidin. Nat. Microbiol..

[18] Winzeler A., Wang J.T. (2013). Purification and culture of retinal ganglion cells from rodents. Cold Spring Harb. Protoc..

[19] Sawamiphak S., Ritter M., Acker-Palmer A. (2010). Preparation of retinal explant cultures to study ex vivo tip endothelial cell responses. Nat. Protoc..

[20] Dyer M.A., Cepko C.L. (2000). Control of Muller glial cell proliferation and activation following retinal injury. Nat. Neurosci..

[21] Ransohoff R.M., Cardona A.E. (2010). The myeloid cells of the central nervous system parenchyma. Nature.

[22] Jover E., Tawk M.Y., Laventie B.J., Poulain B., Prevost G. (2013). Staphylococcal leukotoxins trigger free intracellular Ca(2+) rise in neurones, signalling through acidic stores and activation of store-operated channels. Cell. Microbiol..

[23] Jackson T.L., Antcliff R.J., Hillenkamp J., Marshall J. (2003). Human retinal molecular weight exclusion limit and estimate of species variation. Investig. Ophthalmol. Vis. Sci..

[24] Duarte C.B., Ferreira I.L., Santos P.F., Carvalho A.L., Agostinho P.M., Carvalho A.P. (1998). Glutamate in life and death of retinal amacrine cells. Gen. Pharmacol..

[25] Eberhardt C., Amann B., Feuchtinger A., Hauck S.M., Deeg C.A. (2011). Differential expression of inwardly rectifying K^+^ channels and aquaporins 4 and 5 in autoimmune uveitis indicates misbalance in Muller glial cell-dependent ion and water homeostasis. Glia.

[26] Deeg C.A., Amann B., Lutz K., Hirmer S., Lutterberg K., Kremmer E., Hauck S.M. (2016). Aquaporin 11, a regulator of water efflux at retinal Muller glial cell surface decreases concomitant with immune-mediated gliosis. J. Neuroinflammation.

[27] Reichenbach A., Wurm A., Pannicke T., Iandiev I., Wiedemann P., Bringmann A. (2007). Muller cells as players in retinal degeneration and edema. Graefes Arch. Clin. Exp. Ophthalmol..

[28] Karlstetter M., Scholz R., Rutar M., Wong W.T., Provis J.M., Langmann T. (2015). Retinal microglia: Just bystander or target for therapy?. Prog. Retin. Eye Res..

[29] Hanisch U.K., Kettenmann H. (2007). Microglia: Active sensor and versatile effector cells in the normal and pathologic brain. Nat. Neurosci..

[30] Broderick C., Hoek R.M., Forrester J.V., Liversidge J., Sedgwick J.D., Dick A.D. (2002). Constitutive retinal CD200 expression regulates resident microglia and activation state of inflammatory cells during experimental autoimmune uveoretinitis. Am. J. Pathol..

[31] Zhang Y.K., Zhao L., Wang X., Ma W.X., Lazere A., Qian H.H., Zhang J., Abu-Asab M., Fariss R.N., Roger J.E. (2018). Repopulating retinal microglia restore endogenous organization and function under CX3CL1-CX3CR1 regulation. Sci. Adv..

[32] D’Orazio T.J., Niederkorn J.Y. (1998). A novel role for TGF-beta and IL-10 in the induction of immune privilege. J. Immunol..

[33] Andjelic S., Lumi X., Vereb Z., Josifovska N., Facsko A., Hawlina M., Petrovski G. (2014). A simple method for establishing adherent ex vivo explant cultures from human eye pathologies for use in subsequent calcium imaging and inflammatory studies. J. Immunol. Res..

[34] Werner S., Colin D.A., Coraiola M., Menestrina G., Monteil H., Prévost G. (2002). Retrieving biological activity from LukF-PV mutants combined with different S components implies compatibility between the stem domains of these staphylococcal bicomponent leucotoxins. Infect. Immun..

