# Supplementary material for: Panton-Valentine Leucocidin Proves Direct Neuronal Targeting and Its Early Neuronal and Glial Impacts a Rabbit Retinal Explant Model
Source: Toxins (Basel). 2018 Nov 4;10(11):455. doi: 10.3390/toxins10110455 (PMC6266138; doi:10.3390/toxins10110455)
Supplement: Supplementary file 1 [file toxins-10-00455-s001.pdf]

Supplementary Materials: Panton-Valentine Leucocidin Proves Direct Neuronal Targeting and Its Early Neuronal and Glial Impacts a Rabbit Retinal Explant Model

XuanLi Liu, Michel J Roux, Serge Picaud, Daniel Keller, Arnaud Sauer, Pauline Heitz, Gilles Prévost and David Gaucher

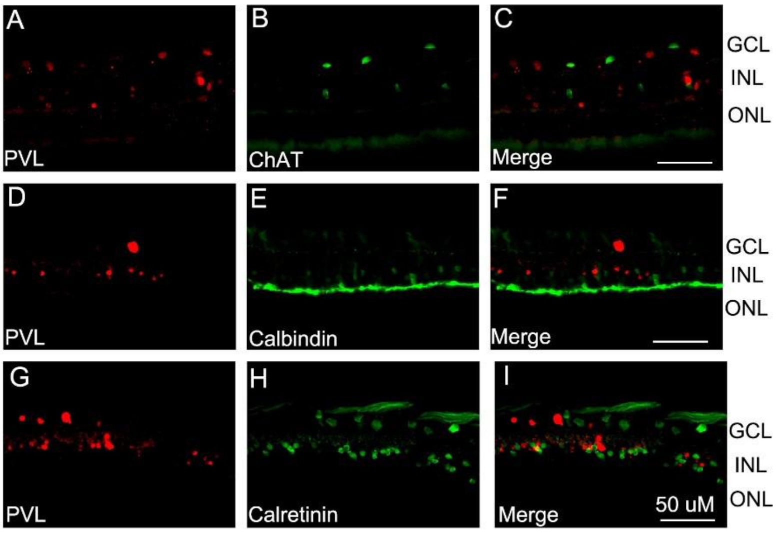

Figure S1. PVL did not colocalize with cholinergic amacrine cells, nor AII amacrine cells; nor calretinin-positive cells (bipolar and horizontal cells) before 4 h after PVL treatment. PVL (red fluorescence) did not colocalize with cholinergic amacrine cells labeled with an anti-ChAT antibody, nor AII amacrine cells labeled with an anti-Calretinin antibody. PVL did not colocalize with calbindin-positive bipolar cells, nor horizontal cells before 4 h after PVL treatment. Abbreviations: PVL, Panton–Valentine leukocidin; ChAT, Anti-Choline Acetyltransferase; GCL, ganglion cell layer; INL, inner nuclear layer; ONL, outer nuclear layer.

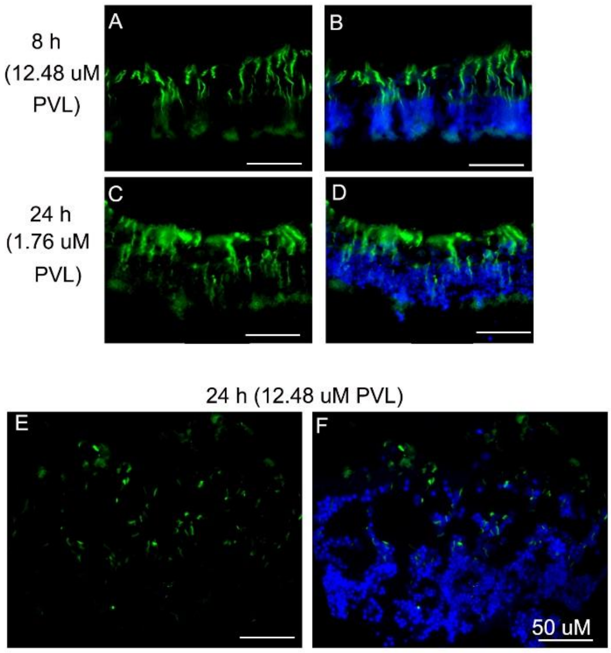

Figure S2. Müller cells were activated and retinal structure was destroyed in PVL-treated explants. Müller cells labeled with an anti-GFAP antibody showed abnormal extension in ONL, and nuclei labeled with Hoechst began to show disordered organization 8 h after PVL treatment (1.76 μM PVL) (**A**,**B**). At 24 h, Müller cell processes were visible in the whole retina, nuclei could not show normal retinal structure in 1.76 μM PVL-treated explants (**C**,**D**). The Müller cells and nuclei were dissolved, retinal structure was destroyed 24 h after PVL treatment (12.48 μM PVL) (**E**,**F**). Abbreviations: PVL, Panton-Valentine leukocidin; GFAP, glial fibrillary acidic protein; ONL, outer nuclear layer.

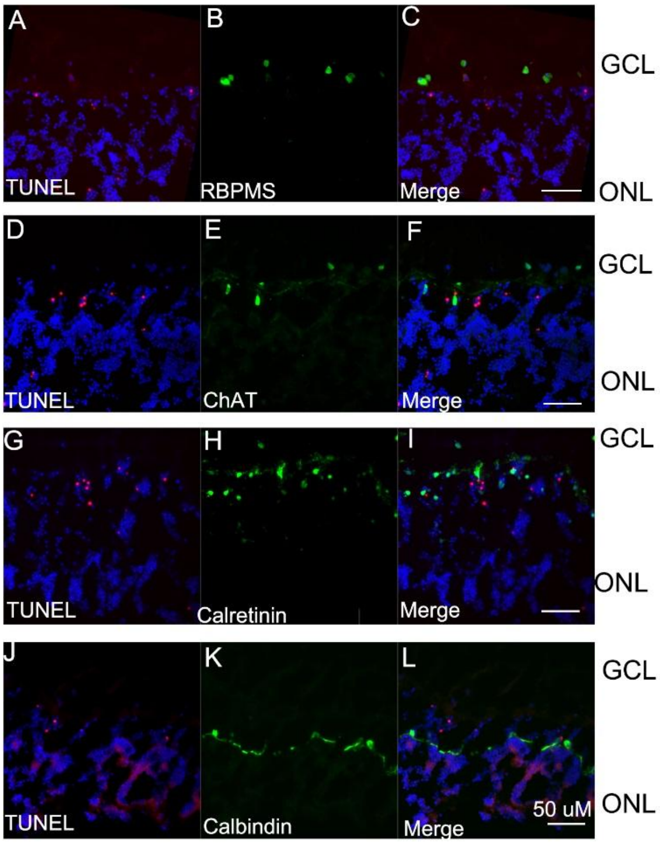

Figure S3. TUNEL positive cells did not colocalized with RGCs, nor cholinergic amacrine cells, nor AII amacrine cells, nor calretinin-positive cells (bipolar and horizontal cells) in PVL-treated explants. The TUNEL positive cells did not colocalized with RGCs labeled with an anti-RBPMS antibody (**A**–**C**), nor with cholinergic amacrine cells labeled with an anti-ChAT antibody (**D**–**F**), nor with AII amacrine cells labeled with an anti-calretinin antibody (**G**–**I**), nor with calbindin-positive bipolar or horizontal cells labeled with an anti-calbindin antibody (**J**–**L**). Abbreviation: PVL, Panton-Valentine leukocidin; TUNEL, terminal deoxynucleotidyl transferase dUTP nick end labeling; RGCs, retinal ganglion cells; RBPMS, RNA-binding protein with multiple splicing; ChAT, Anti-Choline Acetyltransferase; GCL, ganglion cell layer; ONL, outer nuclear layer.
